# Evaluation and Adaption of the Trier Inventory for Chronic Stress (TICS) for Assessment in Competitive Sports

**DOI:** 10.3389/fpsyg.2018.00308

**Published:** 2018-03-13

**Authors:** Jeffrey Sallen, Florian Hirschmann, Christian Herrmann

**Affiliations:** ^1^Faculty of Human Sciences, University of Potsdam, Potsdam, Germany; ^2^Sports Centre, University of Passau, Passau, Germany; ^3^Department for Sport, Exercise and Health, University of Basel, Basel, Switzerland

**Keywords:** chronic stressors, mental health, athletes, stress measurement, Olympic sports, factor analysis, measurement invariance

## Abstract

The demands of a career in competitive sports can lead to chronic stress perception among athletes if there is a non-conformity of requirements and available coping resources. The Trier Inventory for Chronic Stress (TICS) (Schulz et al., 2004) is said to be thoroughly validated. Nevertheless, it has not yet been subjected to a confirmatory factor analysis. The present study aims (1) to evaluate the factorial validity of the TICS within the context of competitive sports and (2) to adapt a short version (TICS-36). The total sample consisted of 564 athletes (age in years: *M* = 19.1, *SD* = 3.70). The factor structure of the original TICS did not adequately fit the present data, whereas the short version presented a satisfactory fit. The results indicate that the TICS-36 is an economical instrument for gathering interpretable information about chronic stress. For assessment in competitive sports with TICS-36, we generated overall and gender-specific norm values.

## Introduction

There is a broad base of evidence-based knowledge concerning stress and its negative impact on health as well as on the competitiveness of humans (Dougall and Baum, 2001; Elfering et al., 2017; Gerber and Schilling, 2017; Siegrist, 2017; Von Dawans and Heinrichs, 2017). Previous research has shown that chronic stress in particular, as opposed to acute stress, is associated with sustained adverse health effects (Schulz and Schlotz, 1999; Dougall and Baum, 2001; Becker et al., 2004; Serido et al., 2004; Fries and Kirschbaum, 2009). While acute stress refers to situations that occur only once and begin or end abruptly, chronic stress is characterized by a creeping onset, frequent recurring stressors with uncontrollable consequences or the absence of effective coping mechanisms, and a usually long-lasting presence. Furthermore, chronic stress may evolve out of an ongoing lack of satisfaction of individual needs (e.g., the need for social support, appreciation, and meaningful tasks), or from non-events (e.g., desired events that do not occur, such as winning medals at important competitions; Pratt and Barling, 1988; Gannon and Pardie, 1989; Wheaton, 1997; Hahn and Smith, 1999).

Following transactional stress concepts (e. g., Lazarus and Folkman, 1984), which are currently favored in psychology and sport psychology (Wippert, 2009; Tamminen and Holt, 2010), subjective perceptions and complex appraisal processes regarding demands, non-events and coping resources play a crucial role in explaining stress. Transactional stress concepts postulate interactions between the individual and their environment, with cognitive processes in the center of the considerations. Stress arises due to a perceived non-conformity of demands and available coping resources (Lazarus and Folkman, 1984; Schwarzer, 2000; Becker, 2006).

There are several approaches to measuring chronic stress and/or chronic stressors via questionnaires (e.g., Cohen et al., 1983; Eckenrode, 1984; Levenstein et al., 1993; Lepore, 1995). None of them was specifically designed for athletes. It is yet to be tested whether these questionnaires are suitable for use in competitive sports. The purpose of this study was to evaluate the Trier Inventory of Chronic Stress (TICS; Schulz and Schlotz, 1999; Schulz et al., 2004) for use in competitive sports. In addition, we are concerned with the adaption, validation, and standardization of a short version of the TICS. But first of all, we want to demonstrate that there are good reasons to take a closer look at chronic stress and stressors of athletes.

The reason for focusing on stress in the research and practice of competitive sports is mainly due to its potential for impairing the competitiveness, well-being, and health of athletes. The possible consequences of stress for athletes include early dropouts from sport careers, sport injuries, mental disorders (e.g., depression), and burnout. A wide range of stressors were identified in studies with athletes including stressors outside of sports as well as sport-specific, competition-specific, and other sport-related stressors (Beckmann and Ehrlenspiel, 2017; Ehrlenspiel et al., 2017; Gustafsson et al., 2017; Sallen, 2017; Tranaeus et al., 2017). The findings that stress research has generated in the field of competitive sports encourage efforts to develop and implement educational psychology interventions for athletes, to help them improve their personal stress resistance and prevent stress-related impairment of their health and performance (Rumbold et al., 2012; Stambulova, 2016; Breslin et al., 2017; Sallen et al., 2017; Schinke et al., 2017). Instruments for stress assessment are of great importance in this context. They are necessary not only for investigating research questions and evaluating effects of stress interventions, but also for monitoring athletes' mental health. Short screening tools make it possible to identify highly stressed athletes with special needs for support at an early stage with only little effort. On the other hand, comprehensive and differentiated stress measurements can help to design problem-centered and individualized interventions. Assessment instruments that can be used in a variety of ways appear particularly attractive.

Overview papers reveal that the topic of stress in competitive sports is geared almost exclusively toward the process of coping with short-term episodes of stress in sport-related settings (mostly competitive situations). Accordingly, the main focus of the counseling work with athletes is on anxiety and acute stress in relation to athletic performance, fatigue, and recovery (Nicholls and Polman, 2007; Thomas et al., 2009; Tamminen and Holt, 2010; Rumbold et al., 2012; Brown and Fletcher, 2016). It is therefore not surprising that most of the instruments used for stress assessment in athletes are focused on these main topics. Although there are many tools for assessing stress in athletes, there is a lack of instruments that are in line with recent stress-related research topics, theoretical perspectives, and approaches to psychosocial support for athletes. This can be seen, for example, in the current trend toward topics that focus on the mental health of athletes (MacIntyre et al., 2017; Schinke et al., 2017). This increasingly requires the use of more general, rather than only sport-specific, stress measurement instruments, such as those mainly used in clinical and health sciences. These instruments were developed for the general population or clinical settings (see below). They should not be applied to athletes without critical reflection on the content and method. It is necessary to adapt or modify them before use. To date, there is hardly any literature on stress measurement in athletes that considers such instruments. This is underpinned by the International Society of Sport Psychology (ISSP), which calls “to further develop existing lines of research on various forms of athletes' mental ill-being, including data on prevalence of mental illness, their sources, and forms of prevention and treatment” (Schinke et al., 2017, p. 12). That is one of the starting points of the present study.

Another starting point that is also suggested by the ISSP position stands and relates with the challenge “to apply a holistic view on athletes' development and their environment to be aware of all range of their current and potential distressors and risk factors” (Schinke et al., 2017, p. 12). Current theoretical positions in sport psychology career research include the holistic lifespan and ecological perspectives. They describe an athlete's career development as multidimensional. Other developmental processes in addition to and partly connected with athletic development are active simultaneously (psychological, psychosocial, academic-vocational, and financial processes). These developmental processes take place in athletic and non-athletic domains of life (Wylleman et al., 2013; Stambulova and Wylleman, 2014). The understanding of athletes as whole persons, who deal with various athletic and non-athletic demands (usually apprised as stressors) from many domains in their everyday life, has thus far received relatively little attention in the development of stress measurement instruments for athletes. If one assumes that overall stress in everyday life is an important factor for athletes' well-being and health, it is important to have well-developed instruments with a broad focus on potential chronic stressors. Such instruments are still rare in health sciences, and even more so in sports science.

From our point of view, the TICS offers a good opportunity to work on some of the described challenges and deficits in sport psychology research and practice. We have selected the TICS for our study, because this instrument has been tested several times on the psychometric properties with predominantly positive results. It has proven to be effective in a clinical and subclinical setting (Buddeberg-Fischer et al., 2010; Petrowski et al., 2012). As the TICS was standardized with a representative sample of the German general population, the test results of male and female individuals of different ages can be interpreted appropriately (Schulz et al., 2004). Some experts in stress research point out that the TICS “is the first instrument that explicitly captures chronic psychosocial stress” (Petrowski et al., 2012, p. 43), independently of specific situations or domains in everyday life. In this respect, the TICS differs from many other stress measurement instruments. It is also important that the TICS covers an age range in which the majority of competitive athletes are located. It can be used by persons aged 14 years and up (Petrowski et al., 2012).

In the context of competitive sports, the TICS has already been used several times for stress assessment in athletes of different ages, types of sports, and competition levels (Nixdorf et al., 2015; Hirschmann, 2017; Richartz and Sallen, 2017). However, the psychometric properties of TICS have not been adequately tested when used on athletes. Consequently, in contrast to previous analyses by Hirschmann (2017), the main focus of the present study is on a confirmatory analysis of the factor structure. Although, the TICS is usually described as a well-validated instrument in scientific literature, there are rarely confirmatory checks on its factorial validity. A confirmatory factor analysis conducted by Petrowski et al. (2012) did not produce optimal results in general population. The factor structure had an unsatisfactory fit with the empirical data and the incremental fit measures (CFI = 0.863, TLI = 0.855, RMSEA = 0.051) remained well below the required cutoff values specified by Hu and Bentler (1999). The reason for this might be the broad construction of the TICS test instrument, which makes it difficult to achieve good fit values. Multidimensional psychological instruments with many items in particular lead to the suppression of significant cross loadings, when used in a confirmatory factor analysis. The model structure can therefore not be represented adequately (Marsh, 2007; Hopwood and Donnellan, 2010; Marsh et al., 2014). Thus, it was hypothesized that, based on item values, this weak point of the TICS also occurs, when it is applied to competitive sports. A reduction of the existing number of items in accordance with the content and psychometric aspects could lead to a smaller number of cross loadings and thus to a better model fit. Therefore, the present study was devoted to the investigation of the psychometric properties of TICS in the field of competitive sports, to optimize the TICS and to adapt an economical short version with high validity. Furthermore, the factor structure was tested for measurement invariance between the genders in a sample of athletes. Based on analyses in general population, it was assumed that the structure is shown to be non-invariant between genders (Schulz et al., 2004; Petrowski et al., 2012).

To interpret results obtained with the newly adapted short version of the TICS, overall and gender-specific norm values had to be generated for individual assessment in athletes.

## Materials and methods

### Instrument

The current German version of the TICS with 57 items was used (Schulz and Schlotz, 1999; Schulz et al., 2004). Each item was rated in a self-assessment on a five-point Likert scale in respect to how often the test subjects had experienced a certain situation or made certain experiences within the last 3 months (0 = never, 1 = rarely, 2 = sometimes, 3 = often, 4 = very often). The intention of the developers of the TICS was to measure the individual experiences with the most important chronic stressors for humans in everyday life. These experiences express the subjectively perceived presence of chronic stressors in terms of their intensity, duration and frequency. Schulz et al. (2004) belief that different chronic stressors have different effects on stress, and that stressor-specific interventions are required to mitigate chronic stress successfully. The items are developed for assessment of nine chronic stressors: (1) *Work Overload* (WO; e.g., “I have too many tasks to perform.”), (2) *Social Overload* (SO; e.g., “I must frequently care for the well-being of others.”), (3) *Pressure to Perform* (PP; e.g., “I have tasks to fulfill that pressure me to prove myself.”), (4) *Work Discontent* (WD; e.g., “There are times when none of my tasks seem meaningful to me.”), (5) *Excessive Demands at Work* (EDW; e.g., “Although I try, I do not fulfill my duties as I should.”), (6) *Lack of Social Recognition* (LSR; e.g., “Although I do my best, my work is not appreciated.”), (7) *Social Tensions* (ST; e.g., “I have unnecessary conflicts with others.”), (8) *Social Isolation* (SI; e.g., “There are times when I have too little contact with other people.”), and (9) *Chronic Worrying* (CW; e.g., “There are times when I worry a lot and cannot stop.”). The terms “work,” “performance,” “tasks,” and “duties” generally refer to performance requirements in everyday life and to requirements from different contexts (e.g., sports, education, occupation, family). The athletes were explicitly referred to this broad understanding of central terms during the oral test instruction and also in the test sheet. The original test items were submitted to the athletes without modifications.

### Samples

We used TICS data from two independent studies with active athletes. Active athletes are defined here as persons who fulfill all of the four following minimum criteria simultaneously: (a) to be training in sports with the aim of improving his/her performance/results; (b) to be actively participating in sport competitions; (c) to be formally registered at a local, regional, or national sport federation as a competitor; and (d) to have sport training and competition as one of his/her major activities or focuses of personal interests (Araújo and Scharhag, 2016). The first study was conducted in 2015 with athletes aged between 14 and 20 years, at three German elite sport schools in the state of Bavaria (sample 1 below; Hirschmann, 2017). In the second study, TICS was used in 2008 for athletes supervised by career advisors at the 19 German Olympic training centers (Hoffmann and Sallen, 2012). This sample (sample 2 below) is widely representative of the group of German athletes between 16 and 44 years. It includes athletes who are professionals in their sport, go to elite sport schools, study at universities, work as trainees in companies, or are pursuing a primary profession alongside their sports career. The samples from the two studies complement each other and form a total sample representing athletes from various Olympic summer sports as well as winter sports. These athletes are distributed across different performance levels—from the regional level (D and D/C elite squads) and the national level (C elite squads) to the international level (A and B elite squads). Details of the samples are listed in Table 1.

**Table 1:** Description of samples with athletes in Olympic sports.

	**Total sample (*N* = 564)**	**Sample 1 (*N* = 169)**	**Sample 2 (*N* = 395)**
**Age (years)**	***M*** **(*****SD*****)**	***M*** **(*****SD*****)**	***M*** **(*****SD*****)**
	19.1 (3.70)	16.3 (1.54)	20.2 (3.72)
**Gender**	***n*** **(%)**	***n*** **(%)**	***n*** **(%)**
Male	312 (55.3)	102 (60.3)	210 (53.2)
Female	252 (44.7)	67 (39.7)	185 (46.8)
**Elite squad level**	***n*** **(%)**	***n*** **(%)**	***n*** **(%)**
A & B squad	164 (29.1)	9 (5.3)	155 (39.2)
C squad	164 (29.1)	28 (16.6)	136 (34.4)
D/C & D squad	236 (41.9)	132 (78.1)	104 (26.3)
**Groups of Olympic sports disciplines**	***n*** **(%)**	***n*** **(%)**	***n*** **(%)**
Endurance sports^a^	170 (30.1)	36 (21.3)	134 (33.9)
Team sports/sports games^b^	90 (16.0)	14 (8.3)	76 (19.2)
Strength and speed-strength sports^c^	145 (25.7)	71 (42.0)	74 (18.7)
Combat sports^d^	65 (11.5)	4 (2.4)	61 (15.4)
Artistic composition sports^e^	38 (6.7)	10 (5.8)	28 (7.1)
Shooting sports^f^	11 (2.0)	0 (0.0)	11 (2.7)
Multi-discipline sports^g^	30 (5.3)	19 (11.2)	11 (2.8)
Anonymous	15 (2.7)	15 (8.9)	0 (0.0)

a e.g., canoeing, running, cycling, rowing, swimming, cross-country skiing;

b e.g., basketball, ice and field hockey, football, volleyball;

c e.g., bobsleigh, weightlifting, athletics (sprinting, jumping, throwing, shot put), skeleton, ski jumping;

d fencing, judo, boxing, wrestling;

e e.g., artistic and rhythmic gymnastics, trampolining, diving;

f e.g., archery, skeet, trap;

g *e.g., biathlon, decathlon, modern pentathlon, Nordic combined*.

### Statistical analysis

We used SPSS® Statistics 22 to process the TICS data and perform a descriptive analysis. Multivariate analyses were performed with Mplus 7.11 (Muthén and Muthén, 2012). The evaluations were carried out in three stages. In the first stage, we checked the TICS instrument for its factorial validity including the data of the first sample. This involved calculating three successive confirmatory and exploratory models: In *model 1*, we examined the nine-factor structure of the TICS instrument in a confirmatory manner according to the test manual (Schulz et al., 2004). This analysis showed an unsatisfactory model fit. We assumed this to be a result of the many items with significant high cross loadings and low factor loadings.

To identify these cross loadings in *model 2*, we therefore calculated an exploratory structural equation model (ESEM) with oblique rotation (GEOMIN) with nine factors (Asparouhov and Muthen, 2009). This approach combines the advantages of the exploratory factor analysis with those of the structural equation model and is recommended for the analysis of the factorial validity of broad multidimensional psychological instruments (Marsh et al., 2014). It tolerates cross loadings, involves the performance of a rotation of the factors, and provides the test statistics of regular structural equation models. After identifying the items with significant high cross loadings, we selected the four test items with the highest factor loadings per factor in *model 2* to adapt a factorially valid and economical short scale. We then used these 36 test items to calculate another exploratory structural equation model in *model 3*.

Due to the good fit indices of *model 3*, our aim in the second stage was to conduct a confirmatory examination of the factorial validity of the short version with the aid of the TICS data from the second sample (*model 4*).

In the *models 5a*−*5c*, we examined the measurement invariance concerning gender, which indicated equality of measurement parameters. Missing measurement invariances would hint at the presence of a differential item functioning (DIF) and would thus restrict the validity of the results on gender differences (Dimitrov, 2006). We realized a sequential procedure. This involved two steps with increasingly stringent nested models: *configural invariance* and *factorial invariance*.

The development of the configural model started with the specification of two independent confirmatory factor analyses for boys and girls, respectively (baseline models 5a). In model 5b, we examined the configural invariance by combining this gender-specific model in a multiple group model. This allowed a model test for boys and girls simultaneously. All parameters were estimated freely. Only the factor structure was equated between boys and girls (Widaman and Reise, 1997; Dimitrov, 2006; Brown, 2015).

The factorial invariance was measured in model 5c, which was based on the configural invariance and restricted the relation between the items and the latent factors. We equated additional factor loadings concerning the boys and girls in the model by constraining the nonstandardized factor loading in model 5b above boys and girls invariantly (Widaman and Reise, 1997; Dimitrov, 2006; Brown, 2015).

Finally, the models 5b and 5c were compared using a difference test. The Mplus module chi-square difference testing for WLSMV was used, as the classical chi^2^-difference tests were not permitted due to the WLSMV estimations (Muthén and Muthén, 2012).

In *model 6* we conducted a confirmatory factor analysis with the covariate gender (male = 0; female = 1) based on *model 4* in order to be able to estimate the differences in the latent factors between boys and girls. Additionally, we requested the modification indices (MI > 10) for the direct effect of the covariate gender in the manifest variables of the test items in order to test again for differential item functioning (DIF).

The *Full Information Maximum Likelihood* (FIML) algorithm implemented in Mplus was used to estimate models in which some of the variables have missing values. Due to chance non-participation in individual test items, there were 25 single missing values in total for all test items (0.26%).

Every analysis was carried out with the help of a probabilistic test model (also: item response theory [IRT]). The manifest (observable) results of the test items are related to the latent factors with a probability mass function (Tenenbaum et al., 2007). The parameter estimation of all estimations was made with the robust weighted least-square (WLSMV) estimation algorithm implemented in Mplus. A two-parameter normal ogive item response theory model (2PNO-IRT) was embedded in an expanded structural equation model. On the basis of the assumption that the continuously distributed latent variables rely on the not continuously distributed observable variables, the relations between the manifest test items and the latent factor are represented by a series of probit regression equations (Muthén et al., 1997). This exploits the benefits of both approaches: the precise estimation of the IRT model as well as the evaluation of the factor structure in the structure equation model by means of the model-fit indices (Herrmann et al., 2015).

In a third step, we calculated a sum score for each stress scale with the 36-item TICS data from the total sample. On the basis of the sum scores, we generated overall and gender-specific norm values (*T-*values) for athletes and indicators for the interpretation of these norm values. The indicators enable the use of the short TICS version for assessment in competitive sports. The procedure is similar to the one described by the TICS developer (Schulz et al., 2004) and follows the recommendations of Lienert and Raatz (1998).

### Procedure

Our work is based on data from two previous studies, which were conducted in conformity with the APA Ethics Code (American Psychological Association, 2017). The first study was examined and approved by the Bavarian State Ministry of Education, Science, and Arts (Hirschmann, 2017), and the second study by the German Federal Institute of Sport Science (Hoffmann and Sallen, 2012). All participating athletes and also the parents of those who were underage provided their informed consent. The Participation was voluntary.

Due to the fact that suitable data from previous studies had already been available for the processing of our research purpose, we have decided not to collect new data and to burden the athletes unnecessarily. We believe that people should only be claimed for research, if research questions cannot be resolved otherwise. The combination and reanalysis of data for evaluation and adaption of the TICS for assessment in competitive sports were first carried out here. This idea arose only after the implementation of the aforementioned studies, which were multidisciplinary, and non-experimental field studies on various topics and issues. Therefore, the reanalysis of data was in line with ethical standards in publishing (American Psychological Association, 2010, pp. 13–15). We assure that the results presented here, have not yet been published, not even in similar form.

## Results

### Factorial validity of the original TICS and adaption of a short version

The examination of the nine-factor structure by confirmatory factor analysis (model 1) with sample 1 showed no satisfactory model fit (χ^2^ = 2173.31, *df* = 1,503, *p* < 0.001, *CFI* = 0.886, *TLI* = 0.879, *RMSEA* = 0.051). The low CFI and TLI values indicated cross loadings of the items, which is why in *model 2* all items were specified to load on all the factors as part of an exploratory structural equation model. This *model 2* achieved a good adjustment to the data (χ^2^ = 1349.25, *df* = 1119, *p* < 0.001, *CFI* = 0.961, *TLI* = 0.944, *RMSEA* = 0.035). Nevertheless, many items showed high cross loadings and low factor loadings (Table 2). The result indicates optimization potential and speaks in favor of economical aspects for the adaption of a short version of the TICS.

**Table 2:** Factor loadings of the models 1–4.

**Stress scale**	**Original TICS item number**	**Model 1 (CFA)**	**Model 2 (ESEM)**									**Model 3 (ESEM)**									**Model 4 (CFA)**
			**F 1**	**F 2**	**F 3**	**F 4**	**F 5**	**F 6**	**F 7**	**F 8**	**F 9**	**F 1**	**F 2**	**F 3**	**F 4**	**F 5**	**F 6**	**F 7**	**F 8**	**F 9**	
1. WO	01	0.67^*^	0.61^*^	0.07	0.03	0.03	0.05	0.18^*^	−0.11	0.00	−0.04										
	04	0.60^*^	0.55^*^	0.01	0.00	0.02	0.13	0.07	0.16^*^	−0.14^*^	−0.12										
	44	0.85^*^	0.64^*^	−0.02	0.00	0.05	0.20^*^	0.17^*^	−0.07	0.07	0.20^*^										
	54	0.85^*^	0.58^*^	0.15^*^	−0.07	0.08	0.08	0.22^*^	−0.02	0.18^*^	0.05										
	17	0.76	0.69^*^	−0.07	0.11	−0.09	0.19^*^	0.05	0.06	0.08	−0.07	0.66^*^	−0.02	0.15	−0.04	0.13	0.04	0.02	0.07	0.13	0.78^*^
	27	0.72^*^	0.64^*^	−0.01	0.22^*^	0.01	−0.06	0.04	0.02	0.06	0.10	0.57^*^	0.06	0.26^*^	0.08	−0.04	0.06	0.01	0.06	0.04	0.66^*^
	38	0.76^*^	0.64^*^	0.10	0.02	0.36^*^	0.05	−0.03	−0.07	−0.01	−0.05	0.59^*^	0.10	0.00	0.47^*^	0.04	0.00	−0.10	−0.05	0.03	0.88^*^
	50	0.81^*^	0.75^*^	0.03	0.01	0.16^*^	−0.02	0.06	0.05	0.06	−0.03	0.70^*^	−0.01	0.04	0.23	0.03	0.11	0.03	0.04	0.04	0.79^*^
2. SO	49	0.50^*^	−0.13	0.12	0.39^*^	0.30^*^	−0.11	0.08	0.17	0.01	−0.15										
	57	0.80^*^	0.16	0.17	0.21	0.05	0.10	0.00	0.53^*^	0.00	−0.25^*^										
	07	0.53^*^	0.19	0.18	0.46^*^	0.10	−0.19	0.00	0.12	0.01	−0.26^*^	0.13	0.31^*^	0.35^*^	0.17	0.00	0.00	0.02	0.02	−0.29^*^	0.36^*^
	19	0.74^*^	0.13	0.78^*^	0.04	−0.02	0.02	0.03	0.02	−0.04	0.04	0.03	0.88^*^	0.00	0.04	0.01	0.00	−0.08	0.01	0.00	0.79^*^
	28	0.73^*^	0.04	0.82^*^	0.00	−0.07	0.00	0.05	0.10	−0.01	0.04	0.00	0.88^*^	−0.03	−0.03	−0.01	0.00	0.07	−0.01	0.03	0.89^*^
	39	0.83^*^	−0.01	0.61^*^	0.21^*^	0.17^*^	0.05	0.07	0.10	0.00	0.00	0.02	0.60^*^	0.17	0.15	0.02	0.05	0.13	−0.05	0.05	0.76^*^
3. PP	08	0.46^*^	0.01	−0.25^*^	0.37^*^	−0.10	−0.03	0.24^*^	0.40^*^	0.02	−0.07										
	12	0.52^*^	−0.23^*^	−0.04	0.27^*^	−0.08	0.21^*^	0.20	0.22	0.35^*^	−0.10										
	14	0.68^*^	0.06	−0.03	0.44^*^	0.48^*^	0.05	−0.14	0.03	−0.04	0.20^*^										
	22	0.56^*^	−0.02	0.05	0.45^*^	−0.01	0.03	0.18	0.06	−0.10	0.24^*^										
	30	0.37^*^	0.02	0.20^*^	0.36^*^	0.03	−0.17^*^	−0.05	−0.03	0.25^*^	−0.01										
	23	0.54^*^	0.01	0.04	0.62^*^	−0.25^*^	0.02	0.33^*^	−0.10	−0.01	0.11	−0.04	0.19	0.54^*^	−0.10	−0.12	0.28^*^	−0.10	−0.01	0.09	0.63^*^
	32	0.55^*^	0.09	−0.16	0.67^*^	0.15	0.23^*^	−0.11	−0.14	−0.09	0.06	0.08	−0.08	0.69^*^	0.16	0.08	−0.12	−0.01	−0.14	0.10	0.53^*^
	40	0.70^*^	−0.10	0.02	0.45^*^	−0.07	0.18^*^	0.19	0.22	0.09	0.05	−0.07	0.17	0.43^*^	−0.15	0.20	0.17	0.21^*^	0.10	−0.01	0.78^*^
	43	0.61^*^	0.26^*^	0.06	0.63^*^	0.04	−0.03	0.05	−0.22	0.00	0.00	0.22	0.02	0.68^*^	0.07	−0.05	0.08	−0.05	−0.04	−0.04	0.58^*^
4. WD	05	0.62^*^	−0.01	0.04	0.05	0.43^*^	−0.04	0.20	0.08	−0.12	0.40^*^										
	10	0.23^*^	−0.56^*^	−0.02	−0.02	0.55^*^	0.08	0.09	−0.07	0.55^*^	−0.08										
	13	0.46^*^	0.15	−0.23^*^	0.06	0.38^*^	0.02	0.08	0.05	−0.12	0.35^*^										
	41	−0.23^*^	−0.64^*^	0.01	−0.07	0.33^*^	−0.16	0.00	−0.04	0.41^*^	−0.01										
	21	0.49^*^	0.05	0.01	−0.15	0.63^*^	−0.16	0.12	0.05	0.14	−0.02	−0.01	0.03	−0.21	0.71^*^	−0.01	0.13	−0.02	0.10	−0.15	0.53^*^
	37	0.65^*^	0.16	−0.01	0.09	0.49^*^	0.05	0.00	−0.03	0.09	0.14	0.09	−0.06	0.10	0.57^*^	0.03	0.01	0.11	0.00	0.08	0.73^*^
	48	0.51^*^	−0.11	−0.02	0.05	0.72^*^	−0.01	−0.11	0.03	0.23^*^	−0.01	−0.10	0.01	0.03	0.54	0.29^*^	−0.07	0.11	0.19	−0.30	0.40^*^
	53	0.78^*^	0.12	0.03	0.02	0.43^*^	−0.04	0.15	0.12	0.28^*^	0.07	0.09	0.06	−0.04	0.46^*^	0.02	0.16	0.12	0.21^*^	0.00	0.71^*^
5. EDW	03	0.66^*^	0.05	0.00	−0.03	0.10	0.44^*^	0.11	0.20^*^	0.00	0.05										
	47	0.70^*^	0.07	−0.06	0.16^*^	0.07	0.50^*^	0.03	0.03	0.16	0.01										
	20	0.81^*^	0.08	0.00	−0.16^*^	0.37^*^	0.70^*^	0.07	0.04	−0.03	−0.04	0.07	0.01	−0.20^*^	0.19	0.66^*^	0.05	−0.02	−0.04	0.28	0.78^*^
	24	0.73^*^	0.14	−0.04	0.07	0.35^*^	0.50^*^	0.00	−0.03	0.03	−0.07	0.11	0.02	0.11	0.14	0.57^*^	0.00	−0.03	0.07	0.02	0.73^*^
	35	0.64^*^	−0.08	−0.02	0.02	0.45^*^	0.63^*^	−0.03	−0.11	−0.02	0.05	−0.11	−0.08	0.05	0.28	0.51^*^	0.01	−0.08	−0.02	0.27	0.52^*^
	55	0.77^*^	−0.02	0.01	−0.03	0.24	0.80^*^	0.04	0.08	−0.04	−0.20	0.00	0.01	−0.04	0.00	0.77^*^	0.02	0.03	−0.03	0.19	0.80^*^
6. LSR	02	0.70^*^	0.15	−0.02	0.04	0.05	−0.06	0.69^*^	0.06	−0.08	0.19^*^	0.04	−0.01	0.02	0.11	−0.04	0.70^*^	0.06	−0.08	0.08	0.77^*^
	18	0.68^*^	−0.03	0.02	−0.02	0.01	0.32^*^	0.64^*^	−0.02	−0.02	−0.01	−0.05	0.08	−0.08	−0.03	0.28^*^	0.65^*^	−0.15	0.02	0.12	0.80^*^
	31	0.88^*^	0.10	0.03	−0.01	0.19^*^	0.16	0.69^*^	0.00	0.05	0.00	0.07	−0.03	0.03	0.00	0.32^*^	0.73^*^	0.09	0.02	−0.04	0.89^*^
	46	0.87^*^	0.10	0.07	0.08	0.26^*^	0.03	0.60^*^	0.06	0.04	0.01	0.09	0.02	0.10	0.15	0.16	0.64^*^	0.10	0.01	−0.07	0.75^*^
7. ST	06	0.68^*^	−0.02	0.07	−0.14	0.12	0.09	−0.03	0.62^*^	−0.04	0.18										
	33	0.70^*^	0.03	0.11	0.10	0.08	0.03	−0.04	0.62^*^	−0.17^*^	0.05										
	15	0.83^*^	0.02	0.05	0.02	0.19	−0.11	−0.03	0.68^*^	0.04	0.21	0.00	0.15	0.04	0.15	−0.01	0.02	0.62^*^	0.05	0.00	0.73^*^
	26	0.69^*^	−0.12	−0.03	−0.01	0.07	−0.02	0.07	0.67^*^	−0.02	0.38^*^	−0.11	−0.03	−0.02	0.09	−0.13	0.09	0.78^*^	−0.13	0.31^*^	0.79^*^
	45	0.79^*^	0.13	−0.06	−0.02	−0.01	0.00	0.03	0.75^*^	0.12	−0.02	0.32^*^	0.00	−0.01	−0.18	0.20	−0.02	0.78^*^	0.04	−0.07	0.77^*^
	52	0.72^*^	0.03	0.04	−0.14	−0.06	0.01	0.03	0.78^*^	0.07	0.03	0.13	0.07	−0.21^*^	0.01	−0.01	0.00	0.72^*^	0.00	0.16	0.71^*^
8. SI	29	0.79^*^	0.05	0.12	0.13	0.03	0.24^*^	−0.04	0.32^*^	0.28^*^	−0.16										
	34	0.60^*^	0.23^*^	0.04	0.07	−0.03	−0.14	0.04	0.00	0.52^*^	0.05										
	11	0.42^*^	−0.34^*^	0.06	0.09	0.02	0.12	0.10	−0.09	0.63^*^	0.02	−0.39^*^	−0.03	0.04	0.00	0.03	0.16	−0.08	0.59^*^	0.16	0.45^*^
	42	0.72^*^	0.05	−0.19^*^	−0.05	0.10	−0.03	−0.05	0.06	0.84^*^	0.04	0.01	−0.20^*^	−0.01	0.07	−0.02	0.03	0.01	0.87^*^	0.01	0.85^*^
	51	0.82	0.17	−0.03	−0.09	−0.02	−0.01	−0.06	0.08	0.85^*^	−0.06	0.17	0.01	−0.08	−0.04	−0.03	−0.02	−0.04	0.91^*^	−0.02	0.89^*^
	56	0.84	0.14	0.00	−0.02	−0.03	0.15	−0.11	0.12	0.70^*^	0.05	0.12	0.00	−0.05	0.02	−0.02	−0.08	0.06	0.68^*^	0.25^*^	0.76^*^
9. CW	09	0.71^*^	−0.20^*^	0.02	0.12	0.13	0.35^*^	0.02	0.06	0.44^*^	0.30^*^	−0.25^*^	0.04	0.09	0.13	0.07	0.00	0.13	0.39^*^	0.42^*^	0.60^*^
	16	0.78	−0.01	0.13	0.13	−0.04	0.53^*^	−0.18^*^	0.14	0.17^*^	0.33^*^	0.00	0.10	0.16	−0.09	0.11	−0.16^*^	0.19^*^	0.14	0.65^*^	0.74^*^
	25	0.83	0.12	0.09	−0.01	−0.11	0.57^*^	0.03	−0.11	0.24^*^	0.44^*^	0.06	−0.01	0.06	−0.02	−0.02	0.05	−0.02	0.19	0.81^*^	0.86^*^
	36	0.84	0.18^*^	0.14	−0.02	−0.06	0.60^*^	−0.04	−0.01	0.07	0.33^*^	0.10	0.09	−0.01	0.07	0.04	−0.01	−0.01	0.05	0.77^*^	0.85^*^

ESEM, exploratory structural equation model; CFA, confirmatory factor analysis; WO, work overload; SO, social overload; PP, pressure to perform; WD, work discontent; EDW, excessive demands at work; LSR, lack of social recognition; ST, social tensions; SI, social isolation; CW, chronic worrying;

* *p < 0.05*.

On the basis of the previous results, we reduced the TICS by 21 items and only retained the four items with the highest loadings per corresponding factor. The reduction had the consequence that the cross loadings in the repeatedly performed exploratory structural equation model were low (χ^2^ = 465.42, *df* = 342, *p* < 0.001, *CFI* = 0.969, *TLI* = 0.944, *RMSEA* = 0.046). The model fit was good and the attribution of the items to the factors was clear (*model 3* in Table 2).

In the following, the adapted short version is called TICS-36.

### Factorial validity and measurement invariance of the TICS-36

The confirmatory analysis of the nine-factor structure of the TICS-36 (*model 4*) using the sample 2 data also had a satisfactory model fit (χ^2^ = 1171.09, *df* = 558, *p* < 0.001, *CFI* = 0.936, *TLI* = 0.928, *RMSEA* = 0.053). The factor loadings were between β = 0.36 and β = 0.89 (Table 2). The latent and manifest correlations between the nine factors laid in the desired mean range of *r* = 0.21 and *r* = 0.68 (Table 3).

**Table 3:** Pearson correlations (below the diagonal) and latent correlations (above the diagonal) of model 4.

**Stress scale**	**1**	**2**	**3**	**4**	**5**	**6**	**7**	**8**	**9**
1. Work overload	1.00	0.48^**^	0.49^**^	0.43^**^	0.47^**^	0.39^**^	0.44^**^	0.32^**^	0.58^**^
2. Social overload	0.44^**^	1.00	0.52^**^	0.41^**^	0.42^**^	0.49^**^	0.63^**^	0.30^**^	0.53^**^
3. Pressure to perform	0.38^**^	0.36^**^	1.00	0.34^**^	0.32^**^	0.37^**^	0.40^**^	0.27^**^	0.43^**^
4. Work discontent	0.29^**^	0.25^**^	0.21^**^	1.00	0.68^**^	0.51^**^	0.54^**^	0.54^**^	0.53^**^
5. Excessive demands at work	0.35^**^	0.30^**^	0.21^**^	0.46^**^	1.00	0.55^**^	0.59^**^	0.47^**^	0.66^**^
6. Lack of social recognition	0.32^**^	0.43^**^	0.27^**^	0.37^**^	0.40^**^	1.00	0.48^**^	0.38^**^	0.39^**^
7. Social tensions	0.33^**^	0.46^**^	0.28^**^	0.37^**^	0.44^**^	0.39^**^	1.00	0.32^**^	0.62^**^
8. Social isolation	0.24^**^	0.29^**^	0.19^**^	0.37^**^	0.36^**^	0.27^**^	0.25^**^	1.00	0.46^**^
9. Chronic worrying	0.46^**^	0.44^**^	0.33^**^	0.37^**^	0.52^**^	0.33^**^	0.39^**^	0.39^**^	1.00

** *p < 0.01*.

Afterwards, we tested whether the relation between the manifest and latent variables (factor loading) for boys and girls were the same (measurement invariance). In *model 5a*, we calculated two separate confirmatory factor analyses for boys (*N* = 210) and girls (*N* = 185). The parameters were released for estimations. Only the factorial structure was kept equal for boys and girls. The two models fit well with the data (boys: χ^2^ = 851.54, *df* = 558, *p* < 0.001, *CFI* = 0.937, *TLI* = 0.929, *RMSEA* = 0.050; girls: χ^2^ = 837.78, *df* = 558, *p* < 0.001, *CFI* = 0.942, *TLI* = 0.934, *RMSEA* = 0.052). This showed that the nine-factorial structure was the same for boys and girls.

These two baseline models for boys and girls were combined to form a multigroup model in *model 5b* (configural invariance). The model fit (χ^2^ = 1778.44, *df* = 1212, *p* < 0.001, *CFI* = 0.940, *TLI* = 0.938, *RMSEA* = 0.049) was good and demonstrated that the factor structure is invariant between boys and girls.

Finally, we tested in multi-group *model 5c* whether the relation between the manifest and latent variables (factor loading) for boys and girls was the same (factorial invariance). The model fit of this model (χ^2^ = 1773.69, *df* = 1239, *p* < 0.001, *CFI* = 0.944, *TLI* = 0.943, *RMSEA* = 0.047) was also good. The difference test between model 5b (variant factor loading) and model 5c (invariant factor loading) showed no significant difference (χ^2^ = 28.94, *df* = 27, *p* = 0.36). This provides the proof that the factor loadings between boys and girls were the same, and thus the measuring model can be assumed to be equal.

In *model 6*, the confirmatory factor analysis with the covariate gender (male = 0; female = 1) also achieved acceptable fit values (χ^2^ = 1197.02, *df* = 585, *p* < 0.001, *CFI* = 0.938, *TLI* = 0.929, *RMSEA* = 0.051) and demonstrated that gender (0 = male, 1 = female) had a significant influence on the latent factors *Work Overload* (β = 0.15, *p* < 0.01), *Social Overload* (β = 0.17, *p* < 0.01), *Excessive Demands at Work* (β = 0.15, *p* = 0.01), *Social Isolation* (β = 0.14, *p* = 0.02), and *Chronic Worrying* (β = 0.36, *p* < 0.01). Female athletes had consistently higher values than male athletes. On the basis of the requested modification indices, no modifications concerning the minimal value (MI > 10) were suggested, which could be rated as additional evidence that there was no DIF between boys and girls.

### Descriptive scale analysis, standardization, and interpretation of the TICS-36

The experiences with chronic stressors in the last 3 months can be represented by sum scores, which are calculated from four items per scale. Each of the nine sum scores has a theoretical value range of 0–16. Table 4 describes the characteristics of the nine TICS-36 scales. In three scales, the stressor values of the study participants in the total sample do not quite reach the upper limit of the possible value range. The strictly conservative Kolmogorov–Smirnov test indicates that the sum scores on all nine scales differ significantly (*p* ≤ 0.001) from a normal distribution. Most scales appear slightly left-skewed. However, given the large sample, an approximate Gaussian normal distribution can be assumed, since almost all distribution values are within the tolerance ranges for skewness (±0.5) and kurtosis (±1.0) recommended by Lienert and Raatz (1998). Due to the reduced number of items, the Cronbach α values of the nine TICS-36 scales (see Table 4) tend to be slightly lower than those of TICS (Hoffmann and Sallen, 2012). However, they are neither well below 0.70 nor above 0.90, so an acceptable internal consistency of all TICS-36 scales may be assumed (Nunnally and Bernstein, 1994; Streiner, 2003).

**Table 4:** Characteristics of TICS-36 with gender differences in stress perception.

**Stress scale**	**Total sample (*N* = 564)**						**Female athletes (*n* = 252)**			**Male athletes (*n* = 312)**		
	**Range of sum score**	***M***	***SD***	**Skewness**	**Kurtosis**	**Cronbach α**	***M***	***SD***	**CI 95%**	***M***	***SD***	**CI 95%**
WO	0–16	7.36	3.29	0.21	−0.18	0.83	7.87	3.40	[7.45, 8.29]	6.95	3.14	[6.60, 7.30]
SO	0–14	4.83	2.84	0.47	−0.08	0.75	5.34	2.77	[4.99, 5.68]	4.42	2.84	[4.11, 4.74]
PP	0–16	8.18	2.78	−0.17	0.22	0.69	8.40	2.67	[8.07, 8.73]	8.00	2.86	[7.68, 8.32]
WD	0–16	5.50	2.70	0.33	0.17	0.68	5.46	2.53	[5.14, 5.77]	5.54	2.82	[5.23, 5.86]
EDW	0–16	5.23	2.71	0.68	0.50	0.78	5.67	2.86	[5.32, 6.03]	4.88	2.54	[4.60, 5.16]
LSR	0–16	5.46	3.22	0.47	0.08	0.83	5.38	3.11	[5.00, 5.77]	5.52	3.31	[5.15, 5.89]
ST	0–14	4.39	2.82	0.54	0.25	0.79	4.55	2.78	[4.20, 4.89]	4.27	2.85	[3.95, 4.59]
SI	0–15	5.48	3.39	0.31	−0.47	0.79	5.85	3.58	[5.41, 6.30]	5.17	3.20	[4.81, 5.53]
CW	0–16	6.73	3.42	0.34	−0.50	0.82	7.92	3.41	[7.50, 8.35]	5.77	3.13	[5.42, 6.12]

*WO, work overload; SO, social overload; PP, pressure to perform; WD, work discontent; EDW, excessive demands at work; LSR, lack of social recognition; ST, social tensions; SI, social isolation; CW, chronic worrying*.

For the evaluation of TICS-36 results, norm values (*T*-values) are provided in Table 5 for athletes. The norm values for the original TICS are not applicable because of changes in the number of items and the expansion of the age range in the representative sample. The evaluation procedure of the TICS-36 does not differ from that of the TICS. First, a sum score is formed from the test values of a person for each of the nine stressors. These sum scores can then be transformed into *T*-values with the data on Table 5. Next, the *T*-values are manually transferred to the original evaluation sheet. The result is an individual profile of chronic stressors in the form of a diagram. Table 6 shows indicators for the interpretation of *T*-values. When interpreting *T*-values, measurement errors must be considered. Each of a person's true *T*-transformed value is added in a confidence interval, whose magnitude depends on the error probability (α = 5%), the standard measurement error, and the scale reliability. In Table 5, *T*-values (only total sample) are marked in italics if the scale-specific confidence interval is completely above the scale mean value (*T* = 50). These *T*-values indicate a significantly above-average relevance of the concerning stressors for the individual. To compare test values of two participants and also to compare two of a person's test values at different times, it is suitable to measure the critical difference between two test values. If the distance between two values regarding a stressor exceeds the scale-specific critical difference, a significant difference is present (α = 5%). With a further measure in Table 6, using the global critical profile difference, it can be determined whether the test values differ significantly (α = 5%) from each other within an individual profile of stressors. This difference is *D*_*crit*_ = 13.5 (Schulz et al., 2004: *D*_*crit*_ = 11.4, Hoffmann and Sallen, 2012: *D*_*crit*_ = 11.8). The profile reliability of the TICS-36 allows a general conclusion about the interpretability of the individual profiles (Lienert and Raatz, 1998, p. 373). It reaches a satisfactory value of _*prof*_ *r*_*tt*_ = 0.66 (Schulz et al., 2004: _*prof*_ *r*_*tt*_ = 0.72; Hoffmann and Sallen, 2012: _*prof*_ *r*_*tt*_ = 0.69).

**Table 5:** Norm values (*T*-values) for total sample (*N* = 564), male athletes (*n* = 312), and female athletes (*n* = 252).

**Raw score**	**WO**			**SO**			**PP**			**WD**			**EDW**			**LSR**			**ST**			**SI**			**CW**		
	**♀♂**	**♀**	**♂**	**♀♂**	**♀**	**♂**	**♀♂**	**♀**	**♂**	**♀♂**	**♀**	**♂**	**♀♂**	**♀**	**♂**	**♀♂**	**♀**	**♂**	**♀♂**	**♀**	**♂**	**♀♂**	**♀**	**♂**	**♀♂**	**♀**	**♂**
0	28	27	28	32	31	34	21	18	22	30	28	30	31	30	31	33	33	33	34	34	35	34	34	34	30	27	32
1	31	30	31	35	34	38	24	22	26	33	32	34	34	34	35	36	36	36	38	37	39	37	36	37	33	30	35
2	34	33	34	37	38	41	28	26	29	37	36	37	38	37	39	39	39	39	42	41	42	40	39	40	36	33	38
3	37	36	37	40	42	45	31	30	33	41	40	41	42	41	43	42	43	42	45	44	46	43	42	43	39	36	41
4	40	39	41	42	45	49	35	33	36	44	44	45	45	44	47	45	46	45	49	48	49	46	45	46	42	38	44
5	43	42	44	45	49	52	39	37	40	48	48	48	49	48	50	49	49	48	52	52	53	49	48	49	45	41	48
6	46	44	47	47	52	56	42	41	43	52	52	52	53	51	54	52	52	51	56	55	56	52	50	53	48	44	51
7	49	47	50	50	56	59	46	45	47	56	56	55	57	55	58	55	55	54	*59*	59	60	54	53	56	51	47	54
8	52	50	53	52	60	63	49	48	50	59	60	59	*60*	58	62	58	58	57	*63*	62	63	57	56	59	54	50	57
9	55	53	57	54	63	66	53	52	53	*63*	64	62	*64*	62	66	*61*	61	61	*66*	66	67	*60*	59	62	57	53	60
10	58	56	60	57	67	70	57	56	57	*67*	68	66	*68*	65	70	*64*	65	64	*70*	70	70	*63*	62	65	*60*	56	64
11	*61*	59	63	59	70	73	60	60	60	*70*	72	69	*71*	69	74	*67*	68	67	*73*	73	74	*66*	64	68	*62*	59	67
12	*64*	62	66	*62*	74	77	*64*	64	64	*74*	76	73	*75*	72	78	*70*	71	70	*77*	77	77	*69*	67	71	*65*	62	70
13	*67*	65	69	*64*	78	80	*67*	67	67	*78*	80	76	*79*	76	82	*73*	74	73	*81*	80	81	*72*	70	74	*68*	65	73
14	*70*	68	72	*67*	81	84	*71*	71	71	*81*	84	80	*82*	79	86	*77*	77	76	*84*	84	84	*75*	73	78	*71*	68	76
15	*74*	71	76	*69*	85	87	*75*	75	74	*85*	88	84	*86*	83	90	*80*	80	79	*88*	88	88	*78*	76	81	*74*	71	80
16	*77*	74	79	*72*	88	91	*78*	79	78	*89*	92	87	*90*	86	94	*83*	83	82	91	91	91	*81*	78	84	*77*	74	83

*(1) WO, work overload; SO, social overload; PP, pressure to perform; WD, work discontent; EDW, excessive demands at work; LSR, lack of social recognition; ST, social tensions; SI, social isolation; CW, chronic worrying; (2) Italic T-values indicate a significantly above-average stress level (α = 5%)*.

**Table 6:** Indicators for the interpretation of TICS-36 results in individual assessment with athletes (*N* = 564).

**Stress scale**	**Standard error**	**Confidence limit of *T*-values**	**Critical difference of two *T*-values**	**Global critical profile difference**
Work overload	4.1	±8.1	11.5	13.5
Social overload	5.0	±9.7	13.7	13.5
Pressure to perform	5.6	±10.9	15.5	13.5
Work discontent	5.7	±11.1	15.7	13.5
Excessive demands at work	4.7	±9.3	13.1	13.5
Lack of social recognition	4.1	±8.1	11.4	13.5
Social tensions	4.5	±8.9	12.6	13.5
Social isolation	4.6	±9.0	12.7	13.5
Chronic worrying	4.2	±8.3	11.7	13.5

## Discussion

The aim of the present study was to conduct a confirmatory analysis of the factor structure of the TICS test instrument by means of a sample of athletes. In addition, we adapted a TICS short form for use in competitive sports on the basis of these results.

In accordance with studies dealing with the factorial validity of multi-dimensional psychological measurement tools with many items, such as the NEO-Five-Factor-Inventory, the confirmatory analysis of the nine-factor structure of the original TICS test instrument did not support its priori structure (Vassend and Skrondal, 1997; Marsh et al., 2010). As expected, the analysis based on sample 1 did not acceptably fit the present data. While the exploratory structural equation model fit the model adequately, some of the test items had low factor loadings as well as high cross loadings. Accordingly, we reduced the number of test items to four per factor to adapt a factorially valid form with a distinct attribution of the items to the factors as well as an economical TICS short version. The re-examination of the 36 test items of the TICS short version using an exploratory structural equation model as well as the confirmatory factor analysis on the basis of sample 2, provided satisfactory model adjustments; factorial validity may be presumed. The invariance test with respect to gender we also conducted, showed no measurement invariance. So the TICS-36 is suitable for illustrating differences in the experiences with chronic stressors between male and female athletes. In line with the literature, the analysis showed consistently higher stress levels for the female athletes (Hoffmann and Sallen, 2012; Britton et al., 2017). Gender differences might be mainly based on a differential interaction of androgens and stress hormones from a biochemical perspective, which leads to a higher stress sensitivity and to an increased vulnerability to the development of stress-related pathology in females (Bangasser et al., 2010). This manifests itself inter alia in differences in the cognitive judgment of the demands of a stressful event that have been found between the genders (Kaiseler et al., 2012). Females tend to assess specific stressors as more severely than males (Tamres et al., 2002). In addition, the higher TICS scores of women on the Chronic Worrying Scale may be associated with their propensity for increased self-reflection (rumination), which is a typical female risk factor in the development of resilience (Ittel and Scheithauer, 2008). Female athletes also generally tend to spend more time on training and schooling (e.g., studying for exams) than male athletes, which can contribute to an increased subjective burden due to work overload (Hirschmann, 2017).

In terms of limitations, an examination of the TICS-36 remains to be conducted, even though the convergent and divergent validities of the original TICS have already been confirmed (Schulz et al., 2004). However, since the factor structure as well as the formulation of the items have remained unchanged, it can be assumed that the correlations with significant external criteria will become apparent in a similar manner as in the original. Furthermore, prognostic validity should be analyzed to allow reliable diagnostic conclusions on chronic stressors in the field of competitive sports. A design with repeated measurements would allow the comparison of factor structures across time and an examination of the stability of the measurement invariance with respect to gender. In addition, neither the TICS nor the TICS-36 can be used to predict the extent to which the test values can be applied to a tolerable limit (Schulz et al., 2004). Since such cutoff values indicated a significant increase in the risk of manifesting physical and psychological symptoms, they could be taken as a basis for identifying risk-takers. Appropriate interventions might help these athletes to mitigate the presence of chronic stressors, adjust the level of chronic stress, and reduce the risk of decreased performance, athlete burnout, or sports dropouts (Isoard-Gautheur et al., 2016).

Despite these limitations, the TICS-36 offers several advantages over to the original TICS. For one thing, it is more practical for sports research and sports practice due to its conciseness. The factor structure of the TICS-36 can be replicated much better, even accounting for possible effects of gender. Since the TICS-36 consists of unchanged items and still has the original nine-factor structure, it may be conceivable that it could replace the original TICS not only in the field of assessment with athletes. It is to be tested to what extent the TICS-36 is also suitable for other groups engaged in competitive sports (coaches, referees) as well as for the general population. Due to its focus on chronic stressors, the TICS-36 is particularly suitable as an assessment tool in psychosocial support services for athletes who are simultaneously pursuing their sports career and an educational/vocational career (so-called dual careers) (EU Expert Group, 2012). The TICS-36 thus enriches the range of high-quality instruments for stress assessment in competitive sports. The norm values generated in this study provide orientation for interpreting results obtained with the TICS-36. The group of adolescent athletes receives much closer consideration in these norm values than in the previous TICS standardization (Schulz et al., 2004; Hoffmann and Sallen, 2012). This creates a basis for assessment in sports with an early onset of peak performance age (e.g., artistic and rhythmic gymnastics).

Finally, we would like to point out that the results achieved with TICS-36 and TICS should be interpreted carefully. As is typical of measurement instruments based on self-reported past experiences, a systematic recall bias cannot be ruled out. Results will always be threatened by the limitation of the individual's memory. Therefore, especially when using the TICS-36 in research contexts, actions to minimize and control recall bias are recommended (Raphael, 1987; Hassan, 2005).

## Author contributions

JS and FH were mainly responsible for the overall conception, design, and analysis of this study and wrote the manuscript in equal parts. CH made a substantial contribution to the implementation of the statistical analysis and the interpretation of the results. The manuscript was read, revised and approved to be published by all authors. All authors agree to be accountable for all aspects of the work and ensure that questions related to the accuracy or integrity of any part of the work are appropriately investigated and resolved.

### Conflict of interest statement

The authors declare that the research was conducted in the absence of any commercial or financial relationships that could be construed as a potential conflict of interest.

## Abbreviations

TICS: Trier Inventory for Chronic Stress
ESEM: Exploratory Structural Equation Modeling
CFA: Confirmatory Factor Analysis
DIF: Differential Item Functioning
MIMIC: Multiple Indicators Multiple Causes
FIML: Full Information Maximum Likelihood
WLSMV: Means-and-Variance-Adjusted-Weighted-Least-Squares
TLI: Tucker-Lewis Index
CFI: Comparative Fit Index
RMSEA: Root Mean Square Error of Approximation.
